# Air Pollution from Global Health to Individual Risk Factor—Is It Time for *Enviropathies* in Everyday Clinical Practice?

**DOI:** 10.3390/ijerph19159595

**Published:** 2022-08-04

**Authors:** Savino Sciascia, Gregory Winston Gilcrease, Lorenzo Roccatello, Dario Padovan, Cristiana Peano, Fulvio Ricceri

**Affiliations:** 1 University Center of Excellence on Nephrologic, Rheumatologic and Rare Diseases (ERK-net, ERN-Reconnect and RITA-ERN Member) with Nephrology and Dialysis Unit and Center of Immuno-Rheumatology and Rare Diseases (CMID), San Giovanni Bosco Hub Hospital, Department of Clinical and Biological Sciences, University of Turin, 10124 Turin, Italy; 2 UNESCO Chair in Sustainable Development and Territory Management, University of Turin, 10124 Turin, Italy; 3 Department of Agricultural, Forest and Food Sciences (DISAFA), University of Turin, 10095 Grugliasco, Italy; 4 Center of Biostatistics, Epidemiology and Public Health, Department of Clinical and Biological Sciences, University of Turin, 10043 Orbassano, Italy

**Keywords:** air pollution, global health, omics

## Abstract

While the link between cardiovascular and respiratory conditions and air pollution is well-known, recent studies provided a growing body of evidence that polluted air, particularly air with high levels of particulate matter with a diameter smaller than 2.5 micrometers (PM 2.5), can have a range of negative impacts on health, both in terms of mortality and morbidity. It is time to emphasize the role of environmental factors as contributory factors or determinants of both global and individual health levels, and to consider them together as a health priority, as enviropathies (meant as pathologies caused, triggered or worsened by environmental exposure). Bringing attention to harmful air pollution exposure has fostered population studies, which developed accurate quantification of environmental exposure in polluted regions, aiding our understanding of the dose-response relationship between pollutants and diseases. Those efforts have influenced local and global health policy strategies. Now we face the challenge of controlling environmental pollution and limiting individual exposure to prevent or avoid serious health risks. Is it time for enviropathies in everyday clinical practice?

## 1. Introduction

Outdoor air pollution caused 3.2 million premature deaths worldwide in 2015 [1]. While the link between cardiovascular [2] and respiratory conditions [3] and air pollution is well-known, recent studies provide a growing body of evidence that polluted air, particularly air with high levels of particulate matter with a diameter smaller than 2.5 micrometers (PM 2.5), can have a range of negative impacts on health, both in terms of mortality and morbidity. A constellation of conditions, including chronic kidney disease progression [4], bone fracture [5], premature birth and low birth weight [6], hypertension [7], psychological distress [8], obesity [9], dysregulated glucose metabolism [10], and inflammatory bowel diseases [11], among others, have been observed with a higher prevalence in polluted areas [12,13,14,15,16]. Moreover, several other pollutants, such as particulate matter with a diameter smaller than 10 micrometers (PM 10), nitrogen dioxide (NO_2_), and nitrogen oxides (NO_x_), have been demonstrated to be associated with mortality in different conditions [17,18,19,20].

The Global Burden of Diseases, Injuries, and Risk Factors Study 2019 estimated that the global deaths attributable to air pollution are 2.92 million (2.53–3.33) in females and 3.75 million (3.31–4.24) in males, corresponding to 11.3% (10.0–12.6) of all female deaths and to 12.2% (11.0–13.4) of all male deaths in 2019. Differences across countries are wide, with higher attributable deaths in those countries with a lower sociodemographic index. When considering the disability-adjusted life-years (DALYs), although a halving in the last 20 years was observed, household air pollution is still responsible for 3.6% (2.7 to 4.6) of DALYs, especially in young people. The burden attributable to air pollution (ambient particulate matter, household air pollution, and ambient ozone pollution) is between 10% and 15% of DALYs in almost all western and eastern sub-Saharan Africa, south Asia, southeast Asia, and most provinces in China [14]. The economic losses attributable to air pollution are estimated around US$ 4.6 trillion (6.2% of global economic output) in 2015, corresponding to around 0.5–1% of the Gross Domestic Product (GDP) of the countries for modern pollutants [21,22,23].

The main limitation of most of these studies is that the collection of air pollution is based on an estimation of the air pollution in the baseline living area and it rarely takes into account residences’ modifications, and it is not systematically directly measured on patients [1,2]. Similarly, indoor pollution is rarely considered in terms of health impact, despite the recent progress in air sensor technologies.

Even if the effect of air pollution is relatively low with respect to other risk factors, its spread across entire populations makes it particularly relevant in terms of the number of attributable morbidities and deaths.

### 1.1. Air Pollution from Global Health to Individual Risk Factor

We need to take effective measures to adapt to our increasing exposure to air pollution. Certainly, different measures of adaptation (including controlling emissions, improving air quality, and avoiding negative health outcomes) are already taking place but in a piecemeal manner. However, a more strategic approach is needed to ensure that timely and effective adaptation measures are taken, ensuring coherency across different sectors and levels of governance. Ultimately, from a physician’s standpoint, adaptation is needed to concretely have an impact on the care of patients.

For example, how many physicians are considering air pollution as a risk factor for the development or worsening of a condition in everyday clinical practice? Do we know any nephrologists investing the exposure to polluted air as a variable impacting on the progression to end-stage renal diseases? Or how many obstetricians are asking their patients if they have been living in an area known to have high rates of air pollution when investigating potential causes of premature birth or low birth weight? More critically, how many hours in medical education curricula are dedicated to exploring the cross-talk between air pollution and diseases’ progression?

Despite increasing knowledge in this field, it is likely that we are only scratching the surface in terms of understanding the problem. Consequently, we are still far from taking action to reduce the role of air pollution in clinical practice. In fact, if the overlap of high pollution and large populations is taking a major toll on public health, then there is currently an unparalleled level of interest in this subject in the general medical population and medical education. This is likely attributed to the lack of knowledge about pollution sources that are responsible for promoting or worsening health conditions on an individual level. An essential step in quantifying the health burden of pollution is to obtain a quantitative estimate of the risk of health outcomes that is causally associated with a pollutant. Causality is based on the evaluation of all available evidence (not only epidemiological toxicology but also clinical studies), explicitly or implicitly using a range of criteria to arrive at a qualitative judgment.

Dr. Austin Bradford Hill, an occupational physician, asked more than 50 years ago the following [24]: “…we see that the event B is associated with the environmental feature A, […]. In what circumstances, can we pass from this observed association to a verdict of causation?” Since then, the strength of association, consistency, specificity, temporality, biological gradient, plausibility, coherence, experiment, and analogy, have since become fundamental tenets of causal inference in epidemiology. Nevertheless, the idea of environmental factors, such as air pollution, still faces obstacles to be incorporated in everyday clinical practice as part of the essential medical information for each patient.

We are all aware that air pollution represents a global health emergency. However, we might still struggle to granulate this concept in practical actions and from an individual perspective. When we arrive at the point where collecting information about air pollution exposure will be part of the routine medical history interrogation, it will be possible to assess how air pollution impacts the development or worsening of diseases. Although air pollution is not directly modifiable or treatable as other conventional individual risk factors (at least to date), this should not preclude detailed information about air pollution exposure becoming part of a patient’s medical history (as it happens with smoking or work exposure).

Thus, developing tailored screening strategies to identify subjects at high risk is an important adaptation factor. Recording the patients’ locational history (i.e., where they have lived) and the length of time they have been living there might represent the first step. The second step is to associate each area with the correspondent level of exposure. Ultimately, this can create a risk scale, computing together the personal susceptibility and the overall air polluted exposure. Potentially, some conditions that have been considered *idiopathic* might be better understood.

### 1.2. Omics Evidence

Data coming from the omics sciences further support the concept that air pollution exposure should be part of the equation when considering how we manage diseases. Recently, a growing body of evidence exploring the link between air pollution, cytotoxicity, component profiling, metabolomics and proteomics is showing that some individuals might be more susceptible to the effects of air pollution [25]. Song et al., when investigating the biological toxicity mechanisms caused by atmospheric fine particles in human lung-bronchial epithelium cells, observed that integrated metabolomics and proteomics analysis can be applied to reveal the significant alterations of many metabolic processes, such as glycolysis, the citric acid cycle, amino acid metabolism, and lipid metabolism [19]. Those kinds of observations are paving the way for a new frontier in the omics sciences: the exposomics [26,27,28,29,30], a new era that has the potential, by using high-throughput techniques, to identify biological signatures and pathways that tend to respond to and interact with environmental exposures. Such information could be applied to design new exposure biomarkers, clarify biological plausibility of observed associations, recognize how diverse exposures may act on common or different pathways, and, ultimately, predict environmental health-related conditions before they become clinically overt. Among others, for instance, recent epigenetic studies have further elucidated the direct biological effects of tobacco use and cancer risk associated with smoking [31].

#### A Global Problem of Multifaced Aspects

The above is even more true in the developing world. Existing estimates of the extent of air pollution-related diseases rely on a small number of studies, most of which are based in high-income countries with relatively low air pollution levels and show considerable methodological heterogeneity. For example, many parts of the United States and Europe have seen substantial improvements in air quality over recent decades as a result of regulatory interventions [32,33,34], with corresponding improvements to public health. However, we still see air pollution disproportionally impacting low-income communities in these developed regions. Additionally, air pollution is a serious issue in other regions around the world, particularly countries in Asia with large populations exposed to poor air quality. Emission levels of several harmful pollutants are expected to increase in Asian cities [35].

While the omics approaches have paved the way with multi-level exposure/assessment studies supporting causality between air pollution and diseases, it is time to emphasize the role of environmental factors as contributory factors or determinants of both global *and* individual health levels, and to consider them together as a health priority, as *enviropathies* (pathologies caused, triggered or worsened by environmental exposure). Bringing attention to harmful air pollution exposure has fostered population studies that have developed accurate quantification of environmental exposure in polluted regions, aiding our understanding of the dose-response relationship between pollutants and diseases. Those efforts have influenced local and global health policy strategies [36]. Now we face the challenge to control environmental pollution and limit individual exposure to prevent or avoid serious health risks. Is it time for *enviropathies* in everyday clinical practice?

## Data Availability

Not applicable.
